# Trophic ecology of glass sponge reefs in the Strait of Georgia, British Columbia

**DOI:** 10.1038/s41598-017-19107-x

**Published:** 2018-01-15

**Authors:** Amanda S. Kahn, Jackson W. F. Chu, Sally P. Leys

**Affiliations:** 1 University of Alberta, Edmonton, AB Canada; 2 Institute of Ocean Sciences, Fisheries and Oceans Canada, Sidney, BC Canada

## Abstract

Sponges link the microbial loop with benthic communities by feeding on bacteria. Glass sponge reefs on the continental shelf of western Canada have extremely high grazing rates, consuming seven times more particulate carbon than can be supplied by vertical flux alone. Unlike many sponges, the reef building species *Aphrocallistes vastus* has no microbial symbionts and removes little dissolved organic carbon. To determine how reef sponges therefore get enough food to sustain such substantial grazing we measured stable carbon and nitrogen isotope signatures of water, sediment and sponge tissues. To ensure samples were temporally associated, we also studied the duration particles were retained in tissues in controlled feeding studies using microscopic beads and ^13^C-labeled bacteria. Although fecal pellets were expelled from sponges within 24 hours of feeding, intact bacteria were still found in tissues and sponge tissues retained elevated ^13^C levels for at least 14 days. These independent lines of evidence suggest that carbon in reef sponge tissues may reflect food consumed from days to weeks earlier. Stable isotope analysis suggests that heterotrophic bacteria ingested by the sponges comes from a confluence of trophic subsidies: from terrestrial and oceanic sources, and also potentially on sediment-borne bacteria resuspended by tidal currents.

## Introduction

Sponges contribute to nutrient cycling by bringing microbial food energy into the larger trophic web^1^. In fact the highest grazing rate known to date is carried out by glass sponges that form globally unique deep water reefs on the continental shelf of western Canada and Alaska^2^. There, filtration of the water by dense stands of sponges removes seven times more carbon than can be provided by vertical flux alone and changes the overlying concentrations of bacteria. Such intense grazing raises the question of how glass sponge reefs get enough bacteria to sustain such high densities. While all sponges phagocytose particulate carbon, whether by filtration or carnivory^3–6^, many also supplement their diet by obtaining carbon fixed by symbionts that take up DOC^7^ or that photosynthesize^8^, or by directly phagocytosing those symbionts^9,10^. Sponges may also use DOC directly if it is sufficiently abundant. However both microbial and eukaryotic symbionts are known to be absent from the tissues of *Aphrocallistes vastus*, the dominant species that forms kilometer-long sponge reefs in the Strait of Georgia^11,12^, and DOC is not part of *A. vastus’* diet^13^.

Glass sponges are one of the few filter-feeding species that are common in the deep ocean even though heterotrophic plankton concentrations are relatively low compared to shallow water (10^5^ vs 10^6^ cells ml^−1^)^14^. They are found on the abyssal plain throughout the world^15–20^ and form dense communities in shallow (<500 m) water in Antarctica and the fjords of New Zealand and western North America^21–26^. Sponge reefs are particularly dense communities of filtration units. In the Strait of Georgia, B.C., they can reach densities of up to 40 individual oscula m^−2^ ^27^. In all of these places it is thought that high dissolved silica concentrations as well as cold and dark water drives their distribution, but in Canada’s fjords the peak abundance of glass sponges coincides with the depth of peak abundance of gelatinous plankton in the water column, a coincidence which was hypothesized might reflect food abundance^25^.

Two obvious potential sources of POC for reef sponges are oceanic, arriving by lateral transport of upwelling-driven phytoplankton blooms and their decomposition^28^ from the Strait of Juan de Fuca^29,30^, and terrestrial, derived from mixing of nutrients from the Fraser River and deep water to produce local bacterioplankton blooms^31^. Experimental work has suggested that feeding through use of current-induced flow^32^ may supplement food uptake from lateral currents, which implicates oceanic rather than terrestrial sources. A third potential food source could be microbes and detritus resuspended in sediments around the sponges by tidally driven currents. Considerable microbial productivity occurs in sediments, which can have microbe concentrations 100 or 1000 times greater than in the water column^33^, and microbial communities from resuspended sediments have been suspected to feed other deep-water suspension feeders^18,34,35^. However the particular density of sponges in the reefs suggests an unusual interaction between sediments and the reef. The sponges are so tall they baffle the currents, generating a natural resuspension event each incoming tide^36^. Any excreted material would also therefore remain local and enhance food to the infaunal communities. Together these could generate an oasis of carbon in an otherwise food-poor region.

To determine the source or sources of trophic subsidies to the reef habitat we studied stable isotope ratios of food sources as well as uptake and excretion of food by the reef sponge *A. vastus*. We hypothesized that reefs receive trophic subsidies from several sources. Understanding the feeding behavior and sources of food may help explain why reefs are found where they are, and can possibly lead to predictions of where other sponge beds may be globally, and why those are good sponge habitats.

## Results

### Retention and release of POM by sponge tissues

**Figure 1:**
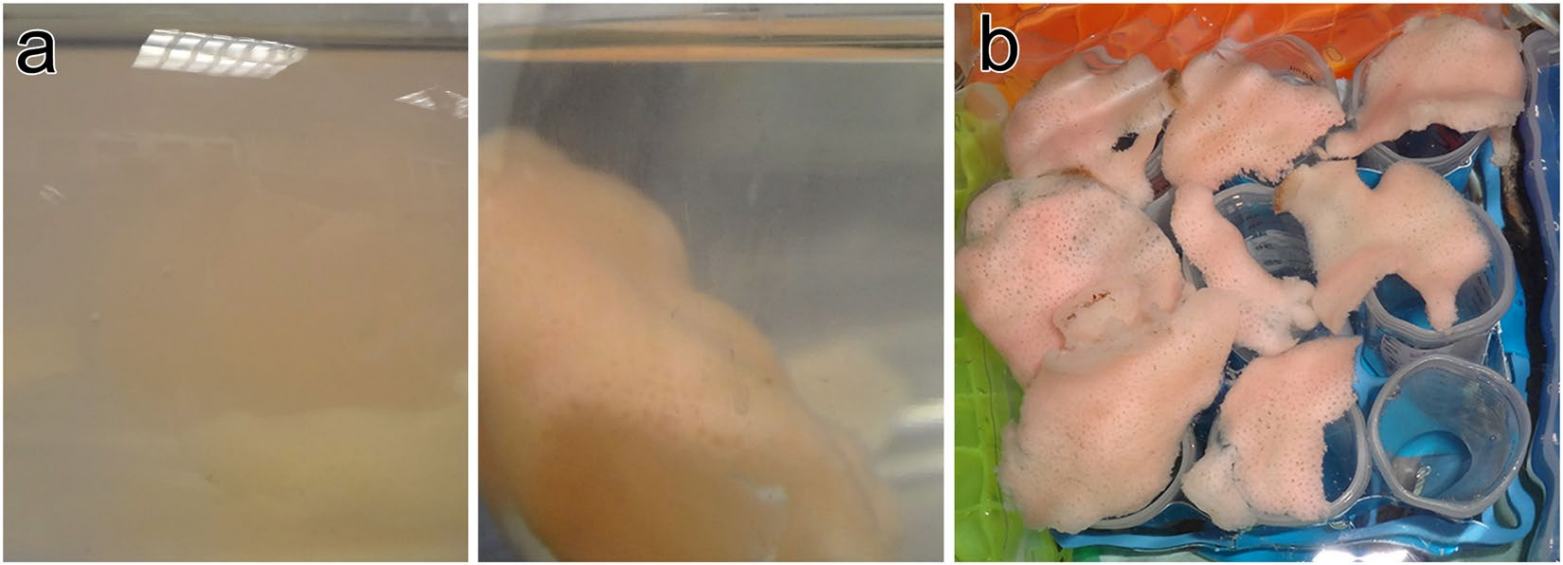
Experimental design to determine how reef sponges captured and excreted food. Pieces of *Aphrocallistes vastus* were incubated in bacteria, 0.1, 0.5, 1.0, and 3.0-µm Fluoresbrite (fluorescent latex) beads (**a**). Water was initially cloudy (left) but became less cloudy as beads were taken up into the tissue (right). Bright pink sponges, colored from eating red fluorescent beads, were left suspended over 50-ml tubes overnight to expel any waste material (**b**). A negative control is visible for the tube in the lower right.

**Figure 2:**
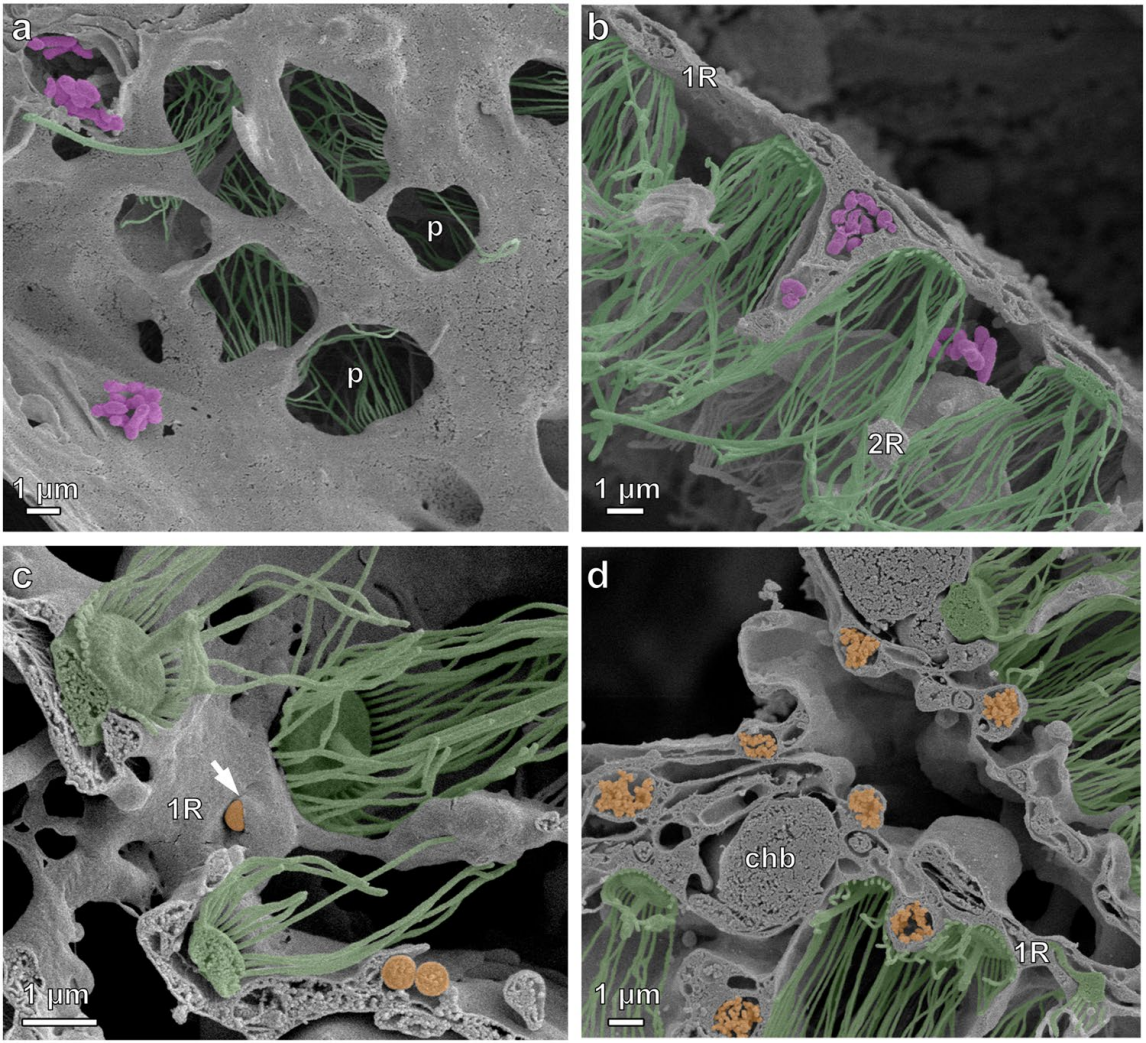
Early stages of particle capture in the primary and secondary reticula of *Aphrocallistes vastus*. (**a**) Bacteria (pseudocolored pink) were visible outside of prosopyles (p) and (**b**) in the spaces between the primary (1 R) and secondary (2 R) reticulum. (**c**) Beads (pseudocolored orange) could be seen being enveloped by small extensions of tissue (arrows) between the collar bodies (pseudocolored green). (**d**) Occurrences of phagocytic vesicles increased as incubations grew longer. Also shown: choanoblasts (chb).

**Figure 3:**
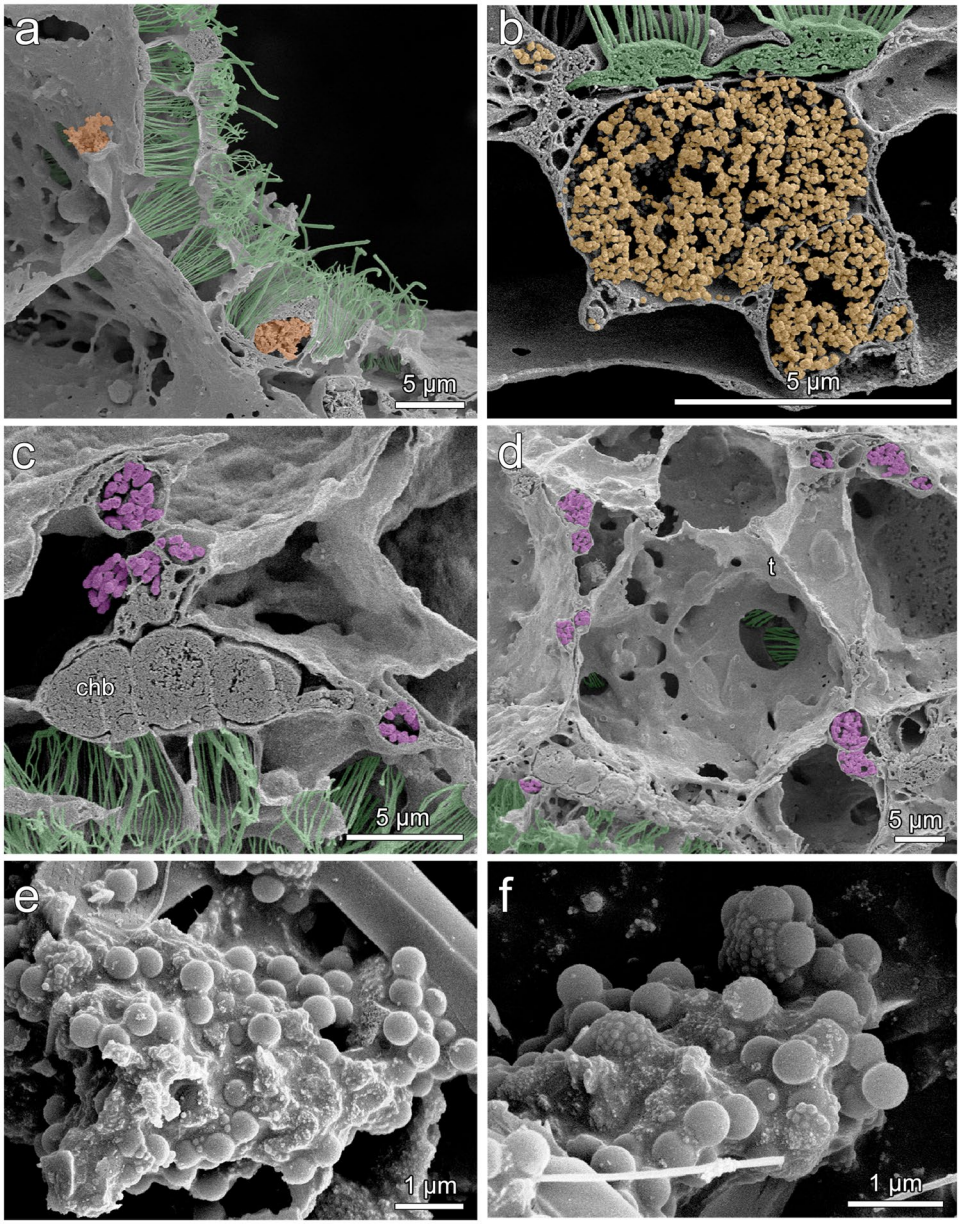
Late stages of particle capture by *Aphrocallistes vastus*. (**a**),(**b**) 0.1 µm beads (pseudocolored orange) were found in phagocytic vesicles below the primary reticulum. (**c**) Over time, phagocytic vesicles filled with undigested bacteria (pseudocolored pink) were visible slightly away from the flagellated chambers behind choanoblasts (chb) and far from the water pumping collar bodies (pseudocolored green). Eventually, packets of undigested bacteria were moved through the trabecular syncytium (t). (**e**),(**f**) Mucus- or membrane-bound clusters of beads gave evidence of fecal pellets from the sponge that are 1000 times greater volume than each bacterial particle consumed.

To determine whether isotope signatures of sponge tissues would reflect conditions of water around the sponges at the time of sampling, we first studied the retention time of particles in sponge tissues in tank experiments from sponges collected during ship cruises to sponge reefs between 2011 and 2014. Sponges cleared the water during 1- and 8-hour incubations with bacteria (Fig. 1a). Sponge tissue became pink during incubations in red fluorescent microspheres; some regions of the tissue were brilliantly colored while others were still the original yellow color of the sponge suggesting different filtration activity by different regions of the sponge. In the shortest incubations both bacteria and 0.1- and 1.0-µm beads were found on and in both the primary and secondary reticula (Fig. 2a–d). While phagocytic vesicles containing beads or bacteria were still visible in the primary and secondary reticula 8 hours after incubation (Fig. 3a and b), vesicles were also found in the trabecular syncytium more distant from chambers (Fig. 3b–d). In all cases bacteria were still whole and undigested in vesicles 8 hours after incubations began (Fig. 3c and d).

We also studied the fate of food eaten by a sponge, as assimilation and excretion, to ensure that food eaten by the sponge is incorporated into tissue, and for how long. Initial attempts to capture excreted material in the field and to detect sponge-derived material in sediments around the sponge were unsuccessful largely due to the low excurrent flow rate from *Aphrocallistes vastus* and general mixing of water around the sponges *in situ*. Therefore we carried out excretion experiments in tanks, and due to shape and size of *Aphrocallistes vastus* we used pieces of the sponge suspended over collection tubes. Excreted material consisted of membrane-bound or mucus-coated clusters of latex beads (Fig. 3e and f); no similar material was found in control tubes without sponge pieces. Mucus-coated waste packets were ellipsoid, 1031 ± 1178 µm^3^ in volume (mean ± standard deviation (s.d.); *n = *10) and were found in larger mucous clusters containing shards of diatom frustules, radiolarian tests, and other unidentifiable detritus. No discarded collar-flagella units or any other recognizable tissues from the sponge were visible.

**Figure 4:**
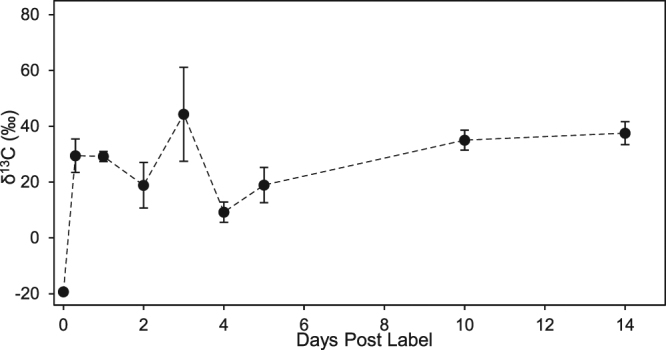
Changes in carbon-13 isotope signatures in an individual of *Aphrocallistes vastus* incubated in water supplemented with ^13^C-labeled bacteria. Black points indicate mean ± standard error δ^13^C of 3 pieces of tissue taken from the same individual.

To study assimilation, a single sponge was fed ^13^C-enriched bacteria and divided into pieces to ensure identical starting material in a pulse-chase experiment. Tissues showed a large spike in δ^13^C after the initial incubation (Fig. 4), and different regions of tissue showed high variability in δ^13^C for the first 5 days post labeling (dpl). After that, δ^13^C values had lower variability 10 and 14 dpl (Levene’s test, F = 0.118, p = 0.051) and were unchanged from the initial spike (F_7,15_ = 2.096, p = 0.109). There was no decrease in δ^13^C in tissues over the 14-day experiment (linear regression, slope = 0.78 ± 0.94 ‰ d^−1^), so the carbon that was assimilated from the bacteria was not excreted or discarded during that time.

### Stable carbon and nitrogen isotope signatures

To study the trophic ecology of the reefs, stable isotope signatures of carbon and nitrogen were measured from sponge tissues of three sponge reefs in the Strait of Georgia and one non-reef location outside of the Strait of Georgia. Carbon isotopes reflect the origin of food (in the case of marine systems, terrestrial or oceanic sources)^37^ and nitrogen isotopes reflect relative trophic position^38^. To determine the carbon and nitrogen isotope signatures of plankton in water immediately adjacent to and therefore filtered by the sponges, we used Niskins on the remotely operated vehicle at two meters above the bottom from the same locations. And to measure the carbon and nitrogen signature of surface sediments around the sponges we sampled the top centimeter of sediment from cores collected using the remotely operated vehicle.

**Figure 5:**
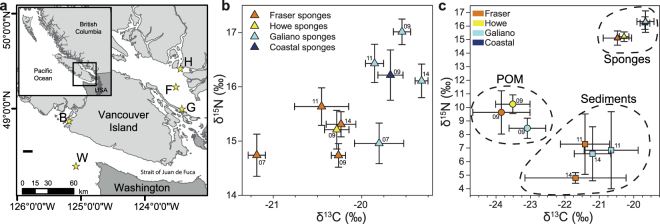
Survey of natural levels of δ^13^C and δ^15^N (‰) of sponges, particulate organic matter, and sediments from several glass sponge reefs. (**a**) Four different locations were sampled to determine natural isotope signatures: Fraser Reef (F), Galiano Reef (G), Howe Sound (H), and sponges found outside of the Strait of Georgia (denoted as ‘coastal’), either in Barkley Sound (B) or off the coast of Washington (W). Map was generated using ESRI ArcMap 10.3^63^ (http://www.esri.com/). (**b**) Stable isotope signatures of sponge tissues from different reefs and across different years (sampling year denoted by the final two digits). (**c**) Stable isotope signatures of particulate organic matter (POM, ●), sponge tissues (▲), and sediments (■) from Fraser Reef (orange), Howe Reef (yellow), Galiano Reef (light blue), and coastal sponges (dark blue). Sponge isotope data collected from the same reef are aggregated across years. Error bars: standard deviation.

Carbon and nitrogen signatures of sponge tissues from three reefs separated by several kilometers in the Strait of Georgia varied between years and reef location (Table 1, Supplemental Table S1). Sponges from Fraser and Howe reefs had significantly lower δ^13^C and δ^15^N ratios than sponges from Galiano reef and those collected from the coastal regions (Table 1, Fig. 5). The variability seen between years and between reefs was less than the difference of the three sample types: sponge tissue, sediments, and POM (Fig. 5). Post hoc Bonferroni comparisons indicated that the mean δ^13^C for sponge tissue (range: −21.31 to −19.25‰) was more similar to sediment (range: −23.80 to −19.73‰; *p* = 0.034) than it was to POM in the water filtered by the sponges (range: −25.22 to −22.49‰; *p* < 0.005). In contrast, δ^15^N ratios in sponge tissue (range: 14.35 to 17.52‰) were more different from those in sediment (range: 4.34 to 10.12‰; *p* < 0.0005) than POM (range: 5.33 to 11.97‰; *p* = 0.042). Large variability meant there was no statistically significant difference between δ^15^N values for POM and sediment, but there was a difference in the δ^13^C values (*p* < 0.0005).

**Table 1:** δ^13^Cand δ^15^N values (‰) measured from Fraser, Galiano, and Howe Reefs, and from two coastal regions: Barkley Sound and Washington coast.

Location	Year	δ^13^C (‰)		δ^15^N (‰)		n
		mean	s.d.	mean	s.d.	
Sponge						
Fraser	2007	−21.19	0.08	14.74	0.31	5
	2009	−20.26	0.11	14.74	0.30	10
	2011	−20.45	0.40	15.64	0.45	9
	2014	−20.23	0.13	15.31	0.19	5
Galiano	2007	−19.80	0.22	14.96	0.30	5
	2009	−19.55	0.14	17.01	0.33	10
	2011	−19.86	0.15	16.43	0.53	11
	2014	−19.32	0.06	16.11	0.25	5
Howe	2009	−20.28	0.19	15.21	0.50	10
Coastal	2008	−19.68	0.13	16.22	0.44	6
POM						
Fraser	2009	−23.84	1.08	9.63	2.08	9
Galiano	2009	−23.09	0.69	8.46	0.97	9
Howe	2009	−23.51	0.70	10.23	0.96	10
Sediments						
Fraser	2011	−21.42	0.58	7.29	1.80	5
	2014	−21.69	1.18	4.80	0.32	5
Galiano	2011	−20.66	0.63	6.84	2.29	5
	2014	−21.69	1.18	4.80	0.32	5

*Aphrocallistes vastus* were sampled between 2007 and 2014 to assess spatial and temporal differences in isotopic signatures. POM was collected as a potential food source for the reefs in 2009, and sediments were sampled first in 2011 to assess whether sponge-derived material (fecal pellets) were traceable, then in 2014 as a potential food source for the reefs.

Quantitative calculation of isotopic fractionation of sediments and POM to the sponge diet and trophic position of the sponges could not be calculated because δ^13^C and δ^15^N ratios for the sponges did not fall between the values of isotope signatures of sediments and POM^39,40^. Qualitatively, POM was the most depleted of ^13^C followed by sediment. Sponge tissue had the lowest depletion of ^13^C.

### Water conditions overlying reef sponges

**Figure 6:**
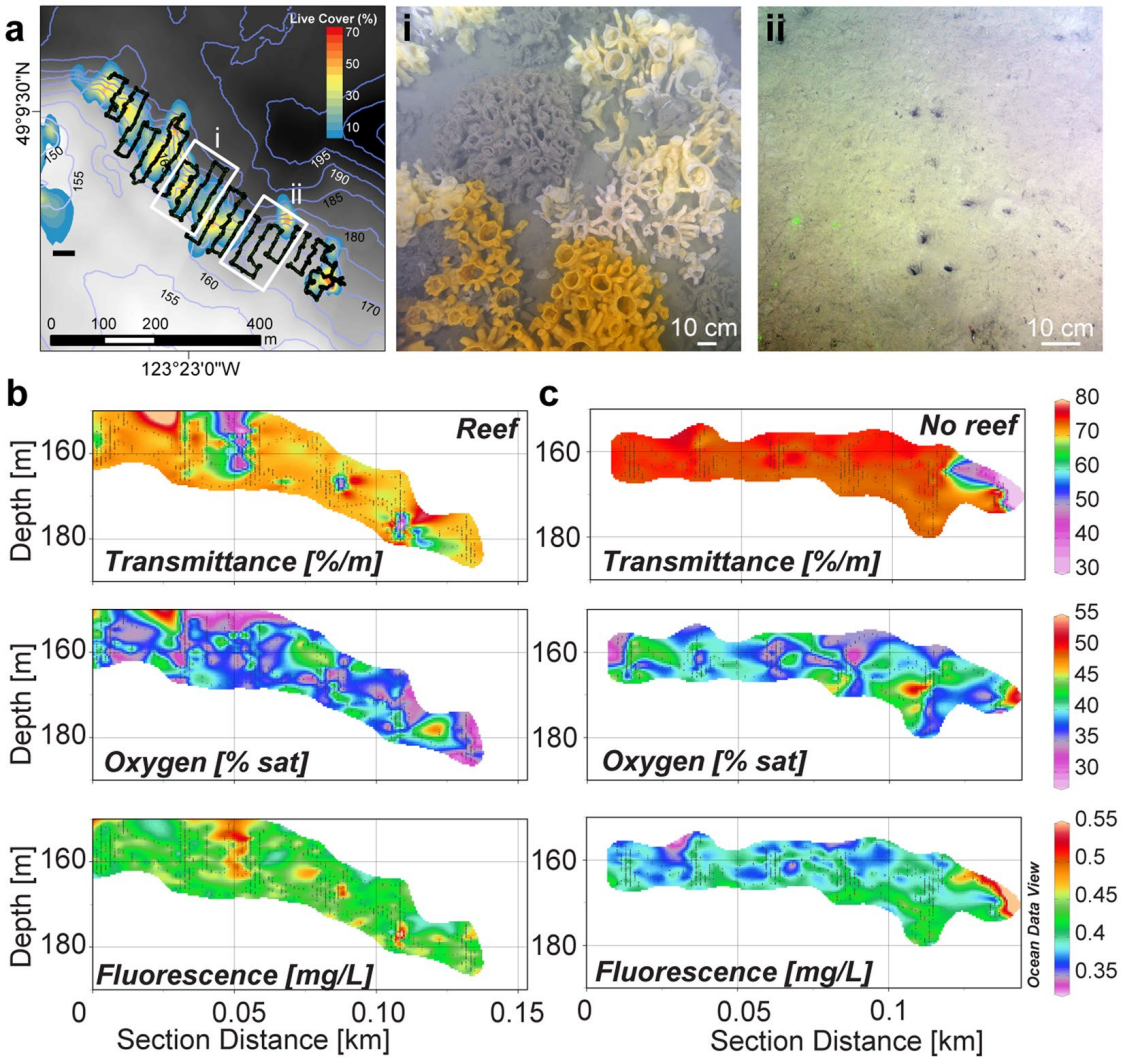
Water conditions overlying reef and non-reef regions at Fraser Ridge sponge reef in the Strait of Georgia. (**a**) Heatmap of sponge density on Fraser Ridge reef showing the survey track. A SeaBird CTD with a transmissometer, oxygen sensor, and fluorometer was mounted on the remotely operated vehicle ROPOS during the survey. Boxes (i) and (ii) indicate the transects across reef and no-reef respectively that were integrated into sections (from SW to NE) for water property data shown in (**b** and **c**). Section A was overlying sponges (i) while Section B was overlying open sediments (ii). Map was generated using ESRI ArcMap 10.3^63^ (http://www.esri.com/).

The water in the sponge reefs was always more turbid than the surrounding water, in particular during flood tides. We used a transmissometer, fluorometer and oxygen sensors on the ROV and carried out transects at two meters above the bottom to survey water properties overlying the reef. Transmittance was lower (it was more turbid) and fluorescence higher in the region overlying sponges than in an adjacent sponge-free patch; water over the sponges was slightly less saturated with oxygen than water where sponges were absent (Fig. 6).

## Discussion

Deep benthic habitats are often dominated by deposit feeders which can feed directly on organic material (‘marine snow’) settled on the seafloor^41,42^. Yet glass sponges are filter feeders that can thrive in oligotrophic deep water. Our previous calculations of the substantial grazing capacity of the glass sponge reefs on Canada’s western coast led us to hypothesize that considerable lateral transport must bring sufficient food to sustain such dense communities^2^. Our study of stable isotope signatures here however, indicates that while some of the food ingested by reef sponges comes from both terrestrial and oceanic sources via lateral transport, it is likely much is also derived via local resuspension of sediment-borne bacteria.

The glass sponges showed long retention of food as carbon. Microscopy of tissues showed intact bacteria at least 24 hours after feeding and ^13^C tracers from bacteria persisted in the sponge tissue for at least two weeks. In contrast, other experiments found that two demosponges (*Haliclona oculata* and *Dysidea avara*) lost ^13^C enrichment after 1–8 days^43^. The long retention of carbon in the glass sponge may reflect the scarcity of bacterial food in deeper waters; we interpret this to mean that the natural isotope signatures of sponges collected *in situ* reflect more than just the last meal of a sponge, but instead food conditions over at least several weeks.

Differences in isotope signatures of sponge tissues at different reefs show interesting patterns that both reflect the circulation of water in the SoG basin and at the same time suggest that different reefs receive different proportions of food imported from either terrestrial or oceanic sources. Fraser and Howe reefs had similar δ^13^C values and these were depleted relative to δ^13^C values of sponges at Galiano Reef and from outside the SoG, suggesting a stronger terrestrial food supply at Fraser and Howe than at Galiano and coastal locations. Freshwater discharge from the Fraser River and Howe Sound flows directly over Fraser and Howe reefs^44^ while sponges at Galiano reef are farther from river outflows and therefore depend more heavily on currents bringing deep oceanic water^30^.

Whereas carbon isotopes are considered to reflect geographic origin of the food, nitrogen isotopes inform on trophic level due to enrichment of the heavier ^15^N isotope through feeding up the food web. Since these reef-building sponges are known to solely derive food from filtration of heterotrophic bacteria we thought the δ^15^N signature might be low, and were surprised to see sponge tissues with consistently high signatures over the years. A meta-analysis of stable isotope signatures in suspension feeders shows a range of δ^15^N values depending on primary food source (lower if mainly consuming primary producers, phytoplankton, higher if consuming secondary consumers, such as small crustaceans), but most suspension feeders have a low trophic level compared to deposit feeders and predators (Supplemental Table 2). Sponges feed on bacteria^5^ which can be either photosynthetic or heterotrophic, and therefore can have different isotope signatures^45^. But since many sponges also fix nitrogen directly via microbial symbionts, the ^15^N fractionation of the sponge tissue is different for high and low microbial abundance sponges, with HMA sponges generally having lower δ^15^N signatures than LMA sponges^46,47^. Heterotrophic bacteria can be especially ^15^N-enriched if they have consumed organic material from high trophic levels or have acted as detritivores by consuming fecal pellets or other exudates. A glass sponge diet in deep water would consist predominantly of heterotrophic bacteria, which may be the primary reason that δ^15^N values of deep sponges are higher than sponges in shallower habitats (average 10.9 vs. 5.2‰, respectively; Supplemental Table 2)^48^. Alternatively, δ^15^N can also become enriched in the water column by microbial metabolic processes such as denitrification^49^ or through exchange from bacteria to protists in the microbial loop^50^, resulting in a depth-related increase in δ^15^N^51^ that may also contribute to higher δ^15^N values in deep sponges compared to shallow sponges.

However other benthic suspension feeders such as bivalves have also been found to have particularly high δ^15^N values and one hypothesis suggests that recycled nutrients and bacteria in resuspended sediments might account for this^35^. Resuspension has been implicated as an important food source for animals in other systems^35,52^ and even for dense glass sponge aggregations in the northern Atlantic, where sediment resuspension by internal waves was thought to be responsible for a vertical shift in distribution of the sponge *Pheronema carpenteri*^18^. Glass sponges at the reefs had a higher δ^15^N than material in the sediments and in POM, supporting the idea that the sponges may receive some trophic subsidy from bacteria in sediment resuspended during tidal changes in addition to microbes from the water column. Experiments using isotopically labeled material to enrich sediments *in situ* (e.g. Jeffreys, *et al*.^53^) will be necessary to determine whether sediment resuspension does provide a true food source for reef sponges.

That reef sponges could capture a lot of bacteria resuspended in sediments is strongly supported by our survey showing increased turbidity and fluorescence over the sponges compared to adjacent areas without sponges. The glass sponge reef at Fraser Ridge experiences semidiurnal tidal surges, each lasting approximately two hours. The relationship glass sponges have with sediment is complicated. Sediments are an integral part of reef-building, and sponges appear well-designed to baffle and trap sediments that cement their bases together^53^. Although high levels of suspended sediments irritate the sponge and cause arrests of the feeding current^54^, these may allow time for processing material ingested with the sediments^55^. Another consideration is whether glass sponges might actually stimulate the growth of fauna in the surrounding sediments with their excretion and later benefit from that productivity. Interestingly, the closest carbon isotope signature to sponge tissue was sediment. Fecal pellets released by *A. vastus*, like those of demosponges^56,57^ were large (1031 ± 1178 µm^3^). Were each to contain only 10% of the carbon from those bacteria as waste, each fecal pellet could supply up to 3.1 µg of carbon^58^ to the adjacent seafloor. With fecal pellets 100 to 1000 times larger than the food consumed, excretion by sponges could be a means of transporting carbon from the microbial loop in the water column into benthic animal food webs.

Glass sponge reefs are only found in coastal waters of the northeastern Pacific. Here we found that the dense sponge populations in the reefs are supported by several sources of food: both new (allochthonous) food imported by strong currents from terrestrial and oceanic sources, and possibly a recycled (autochthonous) food supply as bacteria on resuspended sediments. Tidally driven currents resuspending sediments are essential for supplying sediments to cement the reef structure^53^, but likely also increase bacterial supply in an otherwise food-poor habitat. A greater understanding of near-bottom ocean currents can be useful in predicting the location of sponge beds in both present and past oceans and in evaluating the contribution of sponges as food oases in deep water.

## Methods

To study particle feeding and uptake, twenty-one individuals of *Aphrocallistes vastus* were collected during research cruises on the Canadian Coast Guard Ship *Vector* in 2011 (*n = *11), 2013 (*n = *6), and 2014 (*n = *4) using the remotely operated vehicle ROPOS (http://www.ropos.com/). Sponges were transferred to Bamfield Marine Sciences Centre without removal from seawater (at 9 °C) and once there, maintained in flow-through aquaria that were continually refreshed with water from 30 meters depth. One reef in particular, Fraser Ridge Reef, was also surveyed using a SeaBird SBE 19plus V2 SeaCAT CTD mounted directly on the remotely operated vehicle ROPOS during a survey across the reef (Fig. 6a). The CTD had an oxygen sensor, transmissometer, and fluorometer (WetLabs). Georeferenced CTD data were plotted using Ocean Data View and 150-meter wide sections created to compare characteristics of water overlying reef sponges and water overlying open sediments at Fraser Ridge. Areas with dense sponges and areas with few sponges were identified using mapping data from Chu and Leys^27,59^.

### Particle uptake and excretion

To identify particle capture and feeding, the sponges were cut into 2 × 5 cm pieces. Fragments from the sponges were mixed each year to randomize placement of a sponge into any given feeding or excretion experiment. Sponge fragments were subjected to one of sixteen treatments (n = 5 replicates per treatment) to study particle feeding: 4 incubation lengths (20 minutes, 1 hour, 8 hours, 24 hours) and 4 feeding media (0.1 µm beads, 1.0 µm beads, 3.0 µm beads, and heat-killed bacteria). All five fragments of a given treatment were placed into a 2 L container of seawater and either latex beads (0.1, 0.5, and 1.0-µm diameter; Fluoresbrite microspheres in red, hereafter called beads) or heat-killed *Roseobacter* bacteria were added to a concentration of 2 × 10^6^ particles ml^−1^. Containers were kept in a 9 °C incubator during feeding incubations to maintain constant temperature. After 15 minutes, 1 hour, 8 hours, and 24 hours, several small pieces were preserved for electron microscopy.

To collect excreted material, we tried several methods. Initially, large branches of sponges (approximately 10 × 10 × 2 cm and each with an osculum) were incubated in 2 × 10^6^ beads ml^−1^ overnight, then rinsed in filtered seawater and placed in 3-L containers of filtered seawater for 24 hours. The water in the containers was concentrated on a 0.2-µm Durapore filter and examined under a fluorescence microscope. Although the sponge tissue appeared pink or yellow (the color of the fluorescent beads used), very few (<20) beads were captured on the filter. We then tried placing bead-fed sponges in flowing seawater oriented with oscula facing down into funnels lined with filters. Because of their large size and asymmetrical shape these individuals often fell off of the tubes or tissues abraded into the funnels. Finally, filters pinned across the osculum allowed sponges to be oriented any direction in flowing seawater but any pore size would cause restricted flow from the osculum, considering that excurrent flow rates from these sponges are 1–3 cm s^−1^ and at low pressure^32^. The method from which we report data followed that of Wolfrath and Barthel^56^, which used smaller fragments of sponge. Fragments cut from the same whole sponges were also used here. Randomly selected fragments (3 × 5 cm; n = 24) were immersed in a mixture of 0.1 and 0.5 µm Fluoresbrite fluorescent beads (Polysciences Inc., PA) at a concentration of 2 × 10^6^ beads ml^−1^ for two hours. Treated sponge pieces were rinsed gently in cold seawater and inverted over 50 ml tubes to collect excreted material (Fig. 1a and b). Three control tubes had no sponge. After 24 hours the sponge pieces were carefully removed and the tube contents – material released by the sponge – was allowed to settle for 1 hour. All but 2 ml was removed and the remaining seawater and settled contents were fixed with 1% osmium tetroxide. After 30 minutes, the fixative was changed to a cocktail of 1% osmium tetroxide and 2% glutaraldehyde in 0.45 M sodium acetate (pH 6.4) buffered with 10% sucrose following a protocol modified from Harris and Shaw^60^ optimized for fixation of glass sponge tissue^61^.

Small pieces of tissue that had been exposed to beads and bacteria were preserved at different times post incubation for electron microscopy in the cocktail fixative described above. The fixative was refreshed after 30 minutes, then left overnight at 4 °C. Tissue was dehydrated to 70% ethanol, desilicified in 4% hydrofluoric acid overnight, and stored in 70% ethanol until further processing. Tissue was dehydrated to 100% ethanol, freeze-fractured in liquid nitrogen, critical point dried, and mounted on aluminum stubs. Pieces were gold coated and viewed using a JEOL 6301 F field emission scanning electron microscope. Excreted material was processed following the same method except it was not desilicified or freeze fractured.

### Carbon transport and assimilation

To further track carbon uptake and transport, a single, whole sponge was immersed in seawater supplemented with heat-killed bacteria that had been grown in ^13^C D-glucose as its food source. In brief, Rhodobacteraceae bacteria *Ruegeria* sp. R11 were grown in a shaking incubator (30 °C at 160 rpm) to early log phase in ½ marine broth (24 hours) then re-cultured at a 1:10 inoculum into ½ marine broth supplemented with ^13^C-labeled D-glucose (1 g L^−1^) as a sugar source. Bacteria were cleaned into sterile marine media, concentrated, and heat killed in a 55 °C water bath for 1 hour. Dead cultures were verified by plating the cultures and incubating at 37 °C overnight to ensure that no colonies grew on the plates.

A single sponge 10 × 20 cm – the largest size that could be collected by the ROV – was incubated in 2 × 10^6^ cells ml^−1 13^C-labeled heat-killed bacteria for 8 hours. Three pieces from different regions of the sponge were also rinsed in distilled water and frozen as pre-treatment controls. Triplicate pieces were collected at 1, 2, 3, 4, 5, 10, and 14 days post-labeling (dpl) and unfed control pieces were collected at the conclusion of the experiment to identify any drift in the isotopic signature of the sponge. Each of the samples was rinsed in distilled water, wrapped in foil, and frozen (−80 °C).

All tissue pieces were lyophilized for 24 hours in a Virtis Freezemobile FM25XL lyophilizer and then crushed to a homogeneous powder with an agate mortar and pestle. Precisely 6 ± 0.001 mg dry mass of each tissue powder sample was prepared into tinfoil discs and compacted. Samples were analyzed for δ^13^C and δ^15^N in the Stable Isotope Facility in the Natural Resources Analytical Laboratory at the University of Alberta following internal protocols. In brief, samples were combusted under oxygen, separated chromatographically, and then analyzed using a Continuous Flow Isotope Ratio Mass Spectrometer (CF-IRMS, ThermoFinnigan Delta+ Advantage). Quality control standards from NIST were run for each isotope (for ^13^C: NBS22, LSVEC, and NBS19; for ^15^N: IAEA-N1, IAEA-N2, and IAEA-N3). δ^13^C and δ^15^N values (ppt, ‰) are presented as ratios of the heavy to light isotope of the standard compared to the Pee-Dee Belemnite for δ^13^C and air for δ^15^N (Equation 1).

1 $${{\delta }}^{n}x(\textperthousand )=\frac{({{\delta }}^{n}{x}_{sample}-{{\delta }}^{n}{x}_{std})}{({{\delta }}^{n}{x}_{std})}\times 1000$$

### Carbon sources for the reefs

To trace carbon sources to reef sponges in the Strait of Georgia (SoG), particulate organic matter (POM) from water, sponge tissue, and sediments were collected between 2007 and 2014 during research cruises on the *CCGS Vector* using the remotely operated vehicle ROPOS from glass sponge reefs. Five to ten sponge tissue samples were collected in different locations and in different years (Fig. 5a, Table 1). Five replicate water samples were collected from within the reef using a suction sampler and Niskin bottles mounted on the ROV. Sponge tissue was collected using a suction sampler mounted on the ROV. Sediments (*n* = 5) were collected from among the sponges using push cores deployed by the ROV. Sample sizes for individual years, locations, and sample types are listed in Table 1.

Four liters of each water sample were filtered through pre-weighed, pre-combusted (500 °C for 5 hours) glass fiber filters (GF/F, nominal pore size: 0.7 µm). Filters were rinsed with distilled water, folded into pre-combusted foil packets, and flash frozen in liquid nitrogen. Sponge samples were rinsed three times with distilled water to remove any salts or dissolved carbonates, packed in tinfoil, and flash frozen in liquid nitrogen. Surface sediments from the top of each push core were scraped into two 2-ml cryovials and flash frozen in liquid nitrogen. All samples were transported to the University of Alberta on dry ice and stored in a −80 °C freezer until further processing.

Tissue and filters were lyophilized for 24 hours, sediments for 48 hours in a Virtis Freezemobile FM25XL lyophilizer. Samples from 2007 and 2009 were processed as described by Chu^62^. Samples collected in 2011 and 2014 were ground to a fine powder using an agate mortar and pestle, then loaded into tin capsules (tissue: 6 ± 0.001 mg, sediments: 40 mg) and delivered to the Natural Resources Analytical Laboratory for isotope analysis as described above.

### Data availability statement

Data are available from the University of Alberta Education and Research Archive: 10.7939/R3NS0MB2K.

## Electronic supplementary material

Supplemental Materials
