# Supplementary material for: Trophic ecology of glass sponge reefs in the Strait of Georgia, British Columbia
Source: Sci Rep. 2018 Jan 15;8:756. doi: 10.1038/s41598-017-19107-x (PMC5768768; doi:10.1038/s41598-017-19107-x)
Supplement: Supplementary file 1 — Supplemental Materials [file 41598_2017_19107_MOESM1_ESM.pdf]

# Trophic ecology of glass sponge reefs in the Strait of Georgia, British Columbia

Amanda S Kahn[1], Jackson WF Chu[2], and Sally P Leys[1,*]

[1]University of Alberta, Edmonton, AB, Canada
[2]Institute of Ocean Sciences, Fisheries and Oceans Canada, Sidney, BC, Canada
[*]Corresponding author: sleys@ualberta.ca

**Supplemental Figure 1. Meta-analysis of stable isotope signatures of suspension feeders from shallow to deep water.** In all figures, the stable isotope signature of reef sponges from this study is denoted by a green triangle. A. Stable isotope signatures of various phyla of suspension feeders (Porifera: squares; all other phyla: circles) from shallow habitats (blue, 0-500 m), mid depths (yellow, 500-1000 m), and deep water (red, >1000 m). B. Same as in A, but only Porifera. C. Stable $\delta^{15}N$ signatures of suspension feeders binned to shallow, mid-depth, and deep water (Porifera: shaded circles; all other phyla: open circles).

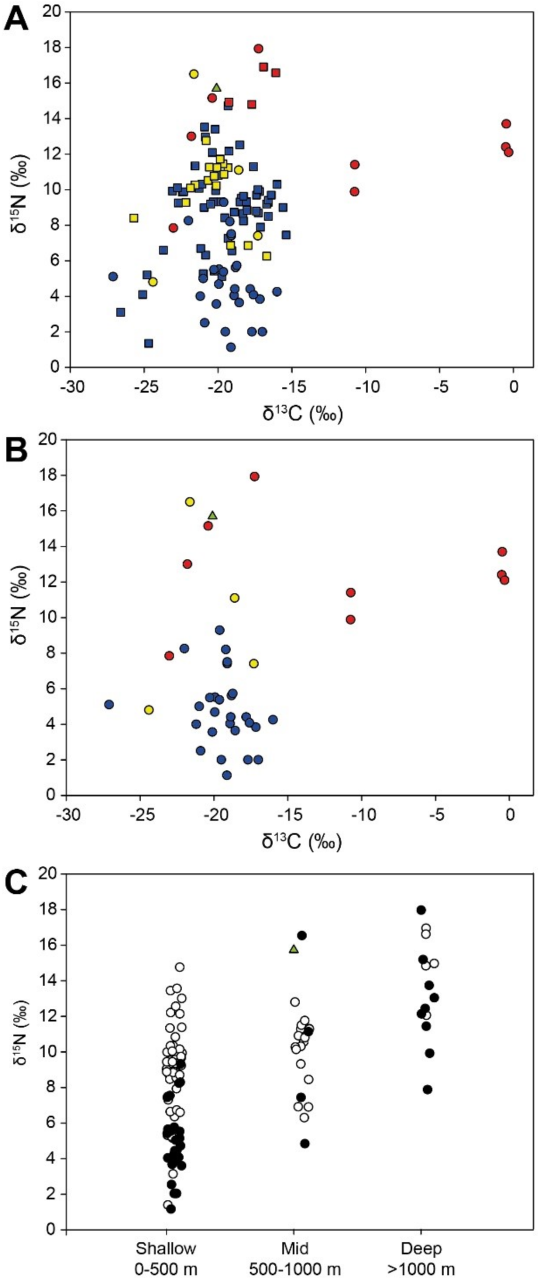

**Supplemental Table S1. Results of post-hoc Bonferroni tests comparing $\delta^{13}C$ and $\delta^{15}N$ values** from sponge tissues collected from (A) Fraser and (B) Galiano reefs in 2007, 2009, 2011, and 2014. (C) Bonferroni post-hoc tests were also used to compare $\delta^{13}C$ and $\delta^{15}N$ values between different sampling locations (Fraser Reef; Howe Reef, Galiano Reef, and coastal sponges from Barkley Sound and off the Washington coastline). Statistically significant p-values ($\alpha$=0.05) are in bold.

A.

Fraser Reef: overall $p < 0.001$

| | 2007 | 2009 | 2011 | 2014 | |
|---|---|---|---|---|---|
| 2007 | - | 1.000 | **0.001** | 0.091 | |
| 2009 | **0.000** | - | **0.000** | **0.035** | **$^{15}N$** |
| 2011 | **0.000** | 0.598 | - | 0.604 | |
| 2014 | **0.000** | 1.000 | 0.713 | - | |
| | **$^{13}C$** | | | | |

B.

Galiano Reef: overall $p < 0.001$

| | 2007 | 2009 | 2011 | 2014 | |
|---|---|---|---|---|---|
| 2007 | - | **0.000** | **0.000** | **0.001** | |
| 2009 | **0.025** | - | **0.015** | **0.002** | **$^{15}N$** |
| 2011 | 1.000 | **0.000** | - | 0.870 | |
| 2014 | **0.000** | 0.073 | **0.000** | - | |
| | **$^{13}C$** | | | | |

C.

By reef (sponge tissue): overall $p < 0.001$

| | Fraser | Galiano | Howe | Pacific | |
|---|---|---|---|---|---|
| Fraser | - | **0.000** | 1.000 | **0.002** | |
| Galiano | **0.000** | - | **0.000** | 1.000 | **$^{15}N$** |
| Howe | 0.569 | **0.000** | - | **0.020** | |
| Coastal | **0.000** | 1.000 | **0.002** | - | |
| | | **$^{13}C$** | | | |

**Supplemental Table 2. Stable carbon and isotope values for organisms identified as filter feeders or suspension feeders.** Organisms were classified as shallow or deep depending on whether the maximum depth was <500 m (shallow) or >500 m (deep). Values of $\delta^{13}C$ and $\delta^{15}N$ were recorded directly from tables or text of original references when possible. If not listed, then values were estimated from graphs. Values written in italics represent the midpoint between the maximum and minimum values for a particular species when no average values were provided. Values that were not specified are labeled with n/s.

| **Species** | **Taxonomic Classification** | **Habitat/Depth (m)** | **$\delta^{13}C$ (‰)** | **$\delta^{15}N$ (‰)** | **Source** |
|---|---|---|---|---|---|
| *Aphrocallistes vastus* | Porifera | 170-1000+ | -20.10 | 15.70 | This study |
| *Perna perna* | Mollusca, Bivalvia | Intertidal | *-15.39* | *7.45* | [1] |
| *Neohelice granulata* | Arthropoda | estuary | -15.6 | 9 | [2] |
| *Laeonereis acuta* | Annelida | estuary | -16.6 | 9.3 | [2] |
| *Mactra isabelleana* | Mollusca, Bivalvia | estuary | -18.3 | 9.4 | [2] |
| *Ostrea puelcheana* | Mollusca, Bivalvia | estuary | -17.6 | 11.3 | [2] |
| *Neomysis americana* | Arthropoda | estuary | -20.4 | 12.1 | [2] |
| *Euphausia superba* | Arthropoda | pelagic | *-24.70* | *1.35* | [3] |
| *Scypha compressa* | Porifera | subtidal | -19.93 | 5.52 | [4] |
| *Tubularia larynx* | Cnidaria | subtidal | -21.17 | 6.69 | [4] |
| *Hiatella arctica* | Mollusca, Bivalvia | subtidal | -19.32 | 7.26 | [4] |
| *Lacuna vincta* | Mollusca, Gastropoda | subtidal | -19.06 | 6.56 | [4] |
| *Bugula* sp. | Bryozoa | subtidal | -21 | 5.27 | [4] |
| *Electra pillosas* | Bryozoa | subtidal | -19.75 | 5.12 | [4] |
| *Botryllus schlosseri* | Chordata, Urochordata | subtidal | -20.84 | 6.32 | [4] |
| *Tunicata* spp. | Chordata, Urochordata | subtidal | -20.26 | 5.42 | [4] |
| *Ophiurae* spp. | Echinodermata, Ophiuroidea | subtidal | -19.54 | 8.42 | [4] |
| *Aplysina cauliformis* | Porifera | 3-4 | -19.5 | 2 | [5] |
| *Aplysina fulva* | Porifera | 2-5 | -21.2 | 4 | [5] |
| *Neopetrosia subtriangularis* | Porifera | 2-5 | -20.9 | 2.5 | [5] |
| *Niphates erecta* | Porifera | 2-5 | -21 | 5 | [5] |
| *Pecten maximus* | Mollusca, Bivalvia | n/s | -16.6 | 8.5 | [6] |
| *Spongia officinalis* | Porifera | 5-20 | *-19.12* | *1.125* | [7] |
| *Halisarca caerulea* | Porifera | 5-20 | *-18.56* | *3.64* | [8] |
| *Clathria raraechelae* | Porifera | 5-20 | -18.91 | 4.03 | [8] |

| | | | | | |
|---|---|---|---|---|---|
| *Chondrilla caribensis* | Porifera | 5-20 | -17.16 | 3.83 | [8] |
| *Chelonaplysilla erecta* | Porifera | 5-20 | -19.94 | 4.68 | [8] |
| *Desmapsamma anchorata* | Porifera | 5-20 | -18.81 | 5.61 | [8] |
| *Scopalina ruetzleri* | Porifera | 5-20 | *-20.28* | *5.49* | [8] |
| *Eurypon laughlini* | Porifera | 5-20 | *-18.73* | *5.725* | [8] |
| *Topsentia ophirhaphidites* | Porifera | 5-20 | -19.63 | 5.37 | [8] |
| *Niphates erecta* | Porifera | 5-20 | *-17.82* | *4.4* | [8] |
| *Callyspongia vaginalis* | Porifera | 5-20 | *-17.60* | *4.08* | [8] |
| *Monanchora arbuscula* | Porifera | 5-20 | *-20.11* | *3.555* | [8] |
| *Phorbas amaranthus* | Porifera | 5-20 | -18.86 | 4.4 | [8] |
| *Ircinia felix* | Porifera | 5-20 | -17 | 2 | [9] |
| *Aplysia cauliformis* | Porifera | 5-20 | -17.7 | 2 | [9] |
| *Niphates erecta* | Porifera | 5-20 | *-16.00* | *4.25* | [9] |
| *Hexactinellid sponges* | Porifera, Hexactinellida | ~4000 | -17.25 | 17.93 | [10] |
| Casperidae | Mollusca, Bivalvia | ~4000 | -16.09 | 16.58 | [10] |
| Capitellidae | Annelida | ~4000 | n/s | 12.02 | [10] |
| Cirripedia 1 | Arthropoda | ~4000 | -19.27 | 14.93 | [10] |
| Cirripedia 2 | Arthropoda | ~4000 | -16.92 | 16.91 | [10] |
| *Culeolus* sp. | Chordata, Urochordata | ~4000 | -17.71 | 14.8 | [10] |
| *Dacrydium* cf. *viteum* | Mollusca, Bivalvia | 625 | -20.12 | 10.23 | [11] |
| Crinoidea unidentified sp. 1 | Echinodermata, Crinoidea | 625 | -17.97 | 6.86 | [11] |
| Porifera | Porifera | 39-54 | *-19.61* | *9.29* | [12] |
| *Astarte borealis* | Mollusca, Bivalvia | 39-54 | -18.39 | n/s | [12] |
| *Clinocardium ciliatum* | Mollusca, Bivalvia | 39-54 | *-18.43* | *9.52* | [12] |
| *Liocyma fluctuosum* | Mollusca, Bivalvia | 39-54 | *-18.52* | *9.31* | [12] |
| *Macoma calcarea* | Mollusca, Bivalvia | 39-54 | *-17.41* | *8.80* | [12] |
| *Ennucula tenuis* | Mollusca, Bivalvia | 39-54 | *-18.33* | *8.67* | [12] |
| *Nuculana radiata* | Mollusca, Bivalvia | 39-54 | *-18.08* | *9.33* | [12] |
| *Pecten* sp. | Mollusca, Bivalvia | 39-54 | -18.53 | 12.52 | [12] |
| *Serripes groenlandicus* | Mollusca, Bivalvia | 39-54 | *-18.28* | *9.63* | [12] |

| | | | | | |
|---|---|---|---|---|---|
| *Yoldia hyperborea* | Mollusca, Bivalvia | 39-54 | *-18.05* | *8.85* | [12] |
| *Alcyonidium gelatinosum anderssoni* | Bryozoa | 39-54 | *-20.33* | *9.32* | [12] |
| *Bowerbankia composita* | Bryozoa | 39-54 | *-18.28* | *8.24* | [12] |
| *Flustra nordenskjoldi* | Bryozoa | 39-54 | -22.69 | 9.26 | [12] |
| *Flustra serrulata* | Bryozoa | 39-54 | -22.41 | 9.86 | [12] |
| *Eucratea loricata* | Bryozoa | 39-54 | *-20.95* | *9.00* | [12] |
| *Dendrobeania* cf *fructicosa* | Bryozoa | 39-54 | -21.29 | 10.08 | [12] |
| Brachiopoda | Brachiopoda | 39-54 | -20.86 | 12.97 | [12] |
| *Boltenia ovifera* | Chordata, Urochordata | 39-54 | -18.91 | 8.74 | [12] |
| *Diplosoma* cf. *listerianum* | Chordata, Urochordata | 39-54 | -23.09 | 9.93 | [12] |
| *Didemnum albidum* | Chordata, Urochordata | 39-54 | -22.33 | 9.89 | [12] |
| Ascidea sp. 1 | Chordata, Urochordata | 39-54 | *-19.80* | *9.32* | [12] |
| cf *Chelyosoma macleayanum* | Chordata, Urochordata | 39-54 | -19.27 | 12.17 | [12] |
| *Astarte borealis* | Mollusca, Bivalvia | 13-58 | -19.32 | 14.72 | [13] |
| *Cyclocardia rjabininae* | Mollusca, Bivalvia | 13-58 | -17.12 | 7.89 | [13] |
| *Alcyonidium vermiculare* | Bryozoa | 13-58 | -17.27 | 8.69 | [13] |
| Unidentified bryozoan | Bryozoa | 13-58 | -22.15 | n/s | [13] |
| *Boltenia echinata* | Chordata, Urochordata | 13-58 | -20.59 | n/s | [13] |
| *Boltenia ovifera* | Chordata, Urochordata | 13-58 | -20.2 | 13.4 | [13] |
| *Chelyosoma* sp. | Chordata, Urochordata | 13-58 | -21.55 | 11.34 | [13] |
| *Molgula* sp. | Chordata, Urochordata | 13-58 | *-21.09* | *10.31* | [13] |
| *Styela* sp. | Chordata, Urochordata | 13-58 | *-20.91* | 13.53 | [13] |
| Unidentified ascidian | Chordata, Urochordata | 13-58 | -20.16 | 9.95 | [13] |
| Unidentified colonial ascidian | Chordata, Urochordata | 13-58 | -22.74 | 10.11 | [13] |
| *Anthomastus grandiflorus* | Cnidaria | 170–1400 | -19.33 | 11.24 | [14] |
| *Duva florida* | Cnidaria | 50–1400 | -19.67 | 11.46 | [14] |

| | | | | | |
|---|---|---|---|---|---|
| *Acanthogorgia armata* | Cnidaria | 170–1400 | -20.59 | 11.27 | [14] |
| *Acanella arbuscular* | Cnidaria | 150–1400 | -20.68 | 10.52 | [14] |
| *Keratoisis ornate* | Cnidaria | 200–1100 | -20.07 | 11.25 | [14] |
| *Paragorgia arborea* | Cnidaria | 370–1300 | -22.18 | 9.28 | [14] |
| *Paramuricea* | Cnidaria | 150–1400 | -19.86 | 11.72 | [14] |
| *Primnoa resedaeformis* | Cnidaria | 160–1100 | -21.56 | 10.27 | [14] |
| *Pennatula* sp. | Cnidaria | 100–1400 | -19.6 | 10.87 | [14] |
| *Bathypathes* sp. | Cnidaria | 750–1300 | -20.27 | 10.76 | [14] |
| *Flabellum alabastrum* | Cnidaria | 220-1400 | -20.81 | 12.76 | [14] |
| *Rhizaxinella pyrifera* | Porifera | >1000 | -24.4 | 4.8 | [15] |
| *Abra longicallus* | Mollusca, Bivalvia | 650-1000 | -16.71 | 6.26 | [16] |
| *Scalpellum scalpellum* | Arthropoda | 650-1000 | -19.15 | 6.87 | [16] |
| *Promachocrinus kerguelensis* | Echinodermata, Crinoidea | ~500 | -25.7 | 8.4 | [17] |
| *Limopsis marionensis* | Mollusca, Bivalvia | ~500 | -21.87 | 10.1 | [17] |
| *Cinachyra antarctica* | Porifera | ~500 | -21.63 | 16.5 | [17] |
| Aggregated Porifera | Porifera | 0 to 170 | *-22.00* | *8.25* | [18] |
| Aggregated Porifera | Porifera | 0 to 30 | -27.1 | 5.1 | [19] |
| *Flustra antarctica* | Bryozoa | n/s | -26.6 | 3.1 | [19] |
| Unidentified bryozoan | Bryozoa | n/s | -25.1 | 4.1 | [19] |
| *Cnemidocarpa verrucosa* | Chordata, Urochordata | n/s | -23.7 | 6.6 | [19] |
| Unidentified tunicate | Chordata, Urochordata | n/s | -24.8 | 5.2 | [19] |
| *Caulophacus arcticus* | Porifera | 2400-3600 | *-10.74* | *11.4* | [20] |
| *Axinellidae* | Porifera | 2400 | -23.02 | 7.84 | [20] |
| *Cladorhiza gelida* | Porifera | 1900-5400 | *-10.75* | *9.885* | [20] |
| *Esperiopsis* sp. | Porifera | 1500-1900 | *-20.39* | *15.15* | [20] |
| *Lissodendoryx complicate* | Porifera | 3100 | -0.48 | 13.7 | [20] |
| *Radiella sol* | Porifera | 3100 | -0.51 | 12.4 | [20] |
| *Tentorium semisuberites* | Porifera | 1900 | -21.8 | 13 | [20] |
| *Thenea abyssorum* | Porifera | 3100 | -0.32 | 12.1 | [20] |

| | | | | | |
|---|---|---|---|---|---|
| *Pachastrella monilifera* | Porifera | 550-700 | -17.3 | 7.4 | [21] |
| *Poecillastra compressa* | Porifera | 550-700 | -18.6 | 11.1 | [21] |
| *Haliclona oculata* | Porifera | 5-8 | -19.1 | 7.4 | [22] |
| *Tethya aurantium* | Porifera | 5-8 | -19.1 | 7.5 | [22] |
| *Dysidea fragilis* | Porifera | 5-8 | -19.2 | 8.2 | [22] |
| *Pisidia longicornis* | Arthropoda | 5-8 | -17.2 | 9.9 | [22] |
| *Crepidula fornicata* | Mollusca, Gastropoda | 5-8 | -16 | 10.3 | [22] |
| *Montacuta ferruginosa* | Mollusca, Bivalvia | 5-8 | -16.7 | 9.2 | [22] |
| *Hiatella arctica* | Mollusca, Bivalvia | 5-8 | -16.6 | 9.4 | [22] |
| *Chlamys varia* | Mollusca, Bivalvia | 5-8 | -17.4 | 9.7 | [22] |
| *Modiolus barbatus* | Mollusca, Bivalvia | 5-8 | -16.4 | 9.7 | [22] |
| *Venus verrucosa* | Mollusca, Bivalvia | 5-8 | -17.3 | 10 | [22] |
| *Thyone fusus* | Echinodermata | 5-8 | -20.5 | 9.2 | [22] |
| *Pyura tesselata* | Chordata, Urochordata | 5-8 | -20 | 10.8 | [22] |

## References


1. Allan, E.L., et al., *Determining spatial changes in the diet of nearshore suspension-feeders along the South African coastline: Stable isotope and fatty acid signatures.* Estuarine, Coastal and Shelf Science, 2010. **87**: p. 463-471.
2. Botto, F., et al., *Origin of resources and trophic pathways in a large SW Atlantic estuary: An evaluation using stable isotopes.* Estuarine, Coastal and Shelf Science, 2011. **92**: p. 70-77.
3. Frazer, T.K., *Stable isotope composition (13C and 15N) of larval krill, Euphausia superba, and two of its potential food sources in winter.* Journal of Plankton Research, 1996. **18**: p. 1413-1426.
4. Fredriksen, S., *Food web studies in a Norwegian kelp forest based δ 13 C and δ 15 N ) analysis on stable isotope ( δ.* Marine Ecology Progress Series, 2003. **260**: p. 71-81.
5. Freeman, C.J. and R.W. Thacker, *Complex interactions between marine sponges and their symbiotic microbial communities.* Limnology and Oceanography, 2011. **56**: p. 1577-1586.
6. Lorrain, A., et al., *Differential y 13 C and y 15 N signatures among scallop tissues : implications for ecology and physiology.* Journal of Experimental Marine Biology and Ecology, 2002. **275**: p. 47-61.
7. Topçu, N.E., et al., *In situ investigation of Spongia officinalis (Demospongiae) particle feeding: Coupling flow cytometry and stable isotope analysis.* Journal of Experimental Marine Biology and Ecology, 2010. **389**(1): p. 61-69.
8. Duyl, F.C., et al., *Coral cavity sponges depend on reef-derived food resources: stable isotope and fatty acid constraints.* Marine Biology, 2011. **158**: p. 1653-1666.
9. Weisz, J.B., et al., *Linking abundance and diversity of sponge-associated microbial communities to metabolic differences in host sponges.* Marine Biology, 2007. **152**(2): p. 475-483.
10. Iken, K., et al., *Food web structure of the benthic community at the Porcupine Abyssal Plain (NE Atlantic): a stable isotope analysis.* Progress In Oceanography, 2001. **50**: p. 383-405.

11. Iken, K., B. Bluhm, and R. Gradinger, *Food web structure in the high Arctic Canada Basin: evidence from δ13C and δ15N analysis.* Polar Biology, 2005. **28**(3): p. 238-249.
12. Iken, K., B. Bluhm, and K. Dunton, *Benthic food-web structure under differing water mass properties in the southern Chukchi Sea.* Deep Sea Research Part II: Topical Studies in Oceanography, 2010. **57**(1): p. 71-85.
13. Feder, H.M., et al., *Benthic food web structure in the southeastern Chukchi Sea: an assessment using δ13C and δ15N analyses.* Polar Biology, 2011. **34**(4): p. 521-532.
14. Sherwood, O.A., et al., *Stable C and N isotopic composition of cold-water corals from the Newfoundland and Labrador continental slope: Examination of trophic, depth and spatial effects.* Deep Sea Research Part I: Oceanographic Research Papers, 2008. **55**(10): p. 1392-1402.
15. Carlier, A., et al., *Heterogeneous energetic pathways and carbon sources on deep eastern Mediterranean cold seep communities.* Marine Biology, 2010. **157**(11): p. 2545-2565.
16. Fanelli, E., et al., *Food web structure of the epibenthic and infaunal invertebrates on the Catalan slope (NW Mediterranean): Evidence from δ13C and δ15N analysis.* Deep Sea Research Part I: Oceanographic Research Papers, 2011. **58**(1): p. 98-109.
17. Mincks, S.L., et al., *Trophic structure on the West Antarctic Peninsula shelf: Detritivory and benthic inertia revealed by δ13C and δ15N analysis.* Deep Sea Research Part II: Topical Studies in Oceanography, 2008. **55**(22): p. 2502-2514.
18. Nyssen, F., et al., *A stable isotope approach to the eastern Weddell Sea trophic web: focus on benthic amphipods.* Polar Biology, 2002. **25**: p. 280-287.
19. Dunton, K.H., *δ15N and δ13C Measurements of Antarctic Peninsula Fauna: Trophic Relationships and Assimilation of Benthic Seaweeds.* American Zoologist, 2001. **41**(1): p. 99-112.
20. Bergmann, M., et al., *Trophic relationships along a bathymetric gradient at the deep-sea observatory HAUSGARTEN.* Deep Sea Research Part I: Oceanographic Research Papers, 2009. **56**(3): p. 408-424.
21. Carlier, A., et al., *Trophic relationships in a deep Mediterranean cold-water coral bank (Santa Maria di Leuca, Ionian Sea).* Marine Ecology Progress Series, 2009. **397**: p. 125-137.
22. Grall, J., et al., *Community structure and food web based on stable isotopes (δ15N and δ13C) analysis of a North Eastern Atlantic maerl bed.* Journal of Experimental Marine Biology and Ecology, 2006. **338**(1): p. 1-15.
